# High-Score US-Suspicious Subcentimeter Thyroid Nodules: What Factors Affect Adequate Sampling of US-Guided Fine-Needle Aspiration Biopsy?

**DOI:** 10.1155/2020/8464623

**Published:** 2020-04-21

**Authors:** Yang Li, Jin Hong Yu, Ping Jie Du, Yu Xie, Sushant Kumar Das, Bing Li, Chuan Zhang

**Affiliations:** ^1^Department of Radiology, The Affiliated Hospital of North Sichuan Medical College, 63 Wenhua Road, Nanchong 637000, Sichuan, China; ^2^Department of Ultrasound, The Affiliated Hospital of North Sichuan Medical College, 63 Wenhua Road, Nanchong 637000, Sichuan, China

## Abstract

**Background:**

Fine-needle aspiration biopsy (FNAB) is diagnostic standard for thyroid nodules. However, the influence of adequate sample rate of US-guided FNAB for subcentimeter thyroid nodules is not known well.

**Objectives:**

To assess the factors affecting adequate sample rate of US-guided FNAB for subcentimeter thyroid nodules.

**Methods:**

Three hundred and forty-nine consecutive US-guided FNAB procedures were performed in 344 patients with subcentimeter thyroid nodules. The adequate sample rate was analyzed for all nodules on the basis of nodule-related and technical factors. The factors affecting adequate sample rate of US-guided FNAB for subcentimeter thyroid nodules were determined by multivariate logistic regression.

**Results:**

The adequate sample rate increased with larger nodules (72.7% for 3–6 mm nodules and 84.9% for 7–10 mm nodules (*P*=0.007)). The adequate sample rate was 63.9%, 81.3%, and 90.6% in nodules with macrocalcifcation, microcalcification, and no calcification, respectively (*P* < 0.001). The adequate sample rate was 71.8% for biopsies performed with a perpendicular needle path and 85.0% with a parallel needle path (*P*=0.004). The significant factors affecting adequate sample rate of US-guided FNAB for subcentimeter thyroid nodules were nodule size (*P* < 0.001; odds ratio (OR) for 7–10 mm nodules was approximately 3.0 times higher than that for 3–6 mm nodules), calcification (*P* < 0.001; OR for nodules without calcification was approximately 5.3 times higher than that for the nodules with macrocalcification), and needle path (*P*=0.044; OR for the use of the parallel needle path was about 1.8 times higher than that for the perpendicular needle path).

**Conclusion:**

Nodule size, calcification, and needle path were the determinants of sample adequacy. The adequate sample rate was higher in larger nodules, in nodules without calcification, and upon using a parallel needle path for biopsy.

## 1. Introduction

Thyroid nodules are a common medical problem. The increasing number of subcentimeter thyroid nodules detected by high-resolution ultrasonography (US) has brought a new problem for management of such subcentimeter nodules. Accurate diagnosis is an important step in the management and treatment of these nodules. US-guided FNAB has been a reliable, safe, and cost-effective method for the differential diagnosis of benign and malignant thyroid nodules [1–4]. The primary purpose of FNAB is to promote the disease management process and determine whether surgery is needed or not. The reported diagnostic accuracy of FNAB is well-established and ranges from 70.2% to 97% [5–10]. However, an inevitable limitation of US-guided FNAB is a nondiagnostic sample, which range in rate from 4.2–40% [8, 11–13]. There are many factors that affect FNAB results, such as nodule size, ultrasound features, sampling technique, needle size, and so on [8, 10, 13–15].

The clinical significance of thyroid papillary microcarcinoma remains controversial; therefore, how to deal with subcentimeter thyroid nodules properly has caused widespread debate [16] The guidelines recommend that FNAB should be considered for nodules less than 1 cm only if these nodules have suspicious ultrasound features, such as hypoechoic nodule, microcalcifications, irregular border, and taller than wide shape [17, 18]. Several studies have been carried out to examine the benefits of US-guided FNAB of subcentimeter thyroid nodules [8, 10, 13, 16, 19], and some experts believe that the combination of suspicious ultrasound features merits referral for US-guided FNAB [8, 19]. However, to our knowledge, there has been no report that has evaluated factors affecting the adequate sample rate of US-guided FNAB for subcentimeter thyroid nodules.

The objective of our study was to retrospectively identify the factors, in relation to lesion characteristics and the biopsy technique, influencing the adequate sample rate of US-guided FNAB for subcentimeter thyroid nodules.

## 2. Materials and Methods

### 2.1. Patients

From January 2016 to October 2018, 3720 thyroid nodules underwent US-guided FNABs in our institution. Among them, 349 nodules (9.4%, 349/3720) in 344 patients (81 men, 263 women; range: 17–78 years, mean age of 45.4 ± 12.3 years) were subcentimeter thyroid nodules. Five patients underwent biopsy for two different nodules. The medical records, ultrasound findings, and cytologic results or/and histologic results in all patients were retrospectively reviewed.

In our practice, the reasons for US-guided FNAB of subcentimeter thyroid nodules were multifactorial. The inclusion criteria were as follows: (a) a thyroid nodule larger than 5 mm with probable malignant assessment on US, (b) a thyroid nodule larger than 5 mm in the patient with high-risk history, and (c) a thyroid nodule in the case of suspected cervical lymph node metastasis [11, 17, 19]. Thyroid nodules were evaluated by using the Thyroid Imaging Reporting and Data System (TI-RADS). Nodules with TI-RADS category 4A and above were considered suspicious lesions. Suspicious malignant US findings included a solid hypoechoic structure, an ill-defined margin, microcalcifications, and a taller than wide shape [19, 20].

### 2.2. US-Guided FNAB Procedure

**Figure 1:**
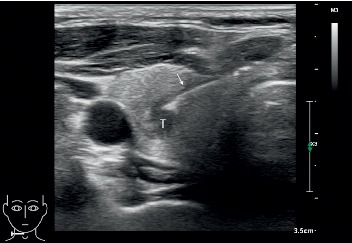
Parallel positioning of the fine needle (arrow) for thyroid nodule (T) biopsy.

**Figure 2:**
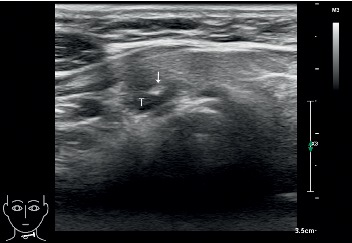
Perpendicular positioning of the fine needle (arrow) for thyroid nodule (T) biopsy.

US-guided FNABs were performed by three interventional radiologists with 6, 8, and 10 years of experience in thyroid biopsy, respectively. All biopsies were performed with US guidance by using a 5–12 MHz linear probe (EPIQ7 or iU 22; Phillips Medical Systems, Bothell, WA, USA). The skin was aseptically prepared and draped, and approximately 1 mL of 1% lidocaine was injected for local anesthesia. US-guided FNABs were performed using a 22- or 23-gauge needle (Hakko, Nagano, Japan) with a free-hand biopsy technique. The biopsy approach for FNAB was used with either a parallel or perpendicular needle path to the probe (Figure 1 and 2). When the needle tip had reached the nodule, tissue samples were collected by using 6 to 7 to-and-fro needle motions over 5–10 seconds within the nodule, and according to the features of thyroid nodules, a combination of the aspiration and the capillary techniques was used. A total of 4 to 6 slides were prepared from the 3 to 4 passes for each of the nodules being biopsied. The specimens were immediately smeared on the glass slides band and then fixed with 95% alcohol.

### 2.3. Cytologic Diagnosis

The specimens were classified as follows according to the Bethesda categories: (I) nondiagnostic; (II) benign; (III) atypia or follicular lesion of undetermined significance (AUS/FLUS); (IV) follicular neoplasm or suspicious for follicular neoplasm (FN/SFN); (V) suspicious for malignancy (SM); and (VI) malignant [21]. Categories II–VI were interpreted as diagnostic results.

The final diagnoses of malignant nodules (including SM) were confirmed by surgery. The final diagnoses of benign nodules were also confirmed by surgery, with clinical and imaging follow-up of at least 1 year.

### 2.4. Data Collection

Nodule-related factors included nodule size, nodule location, and ultrasound features. Nodule size was measured along the maximal diameter. Ultrasound features for the nodules were described for the following features: internal composition (solid, mixed, or cystic), shape (ovoid to round, taller than wide, or irregular), margin (defined or ill-defined), echogenicity (hypoechoic, isoechoic, or hyperechoic), and the presence of microcalcifcation or macrocalcifcation [19, 20]. Technical factors included needle path, individual radiologist, and biopsy needle gauge.

### 2.5. Statistical Analysis

The chi-square test or Fisher exact test was used for the categorical variables. The two-tailed *t*-test was used to analyze the mean of a continuous variable. Multivariate logistic regression analysis was performed to select statistically significant variables with *P* < 0.05 in univariate analysis. Statistical analyses were performed by using SPSS software（version 17, SPSS Inc, Chicago, IL）, and a *P* < 0.05 was considered as a statistically significant difference.

## 3. Results

### 3.1. Cytological Results and Surgical Results

**Figure 3:**
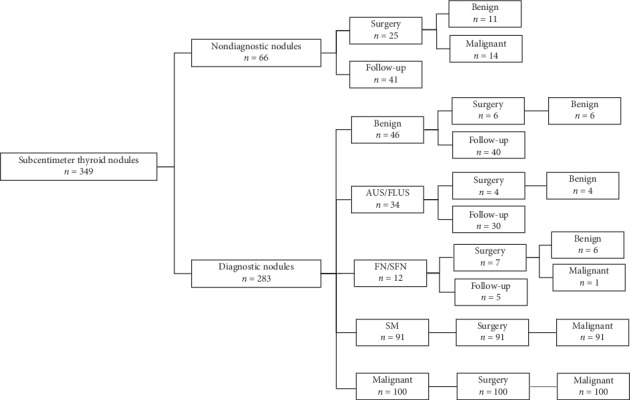
Flowchart showing the study population of 344 patients with 349 subcentimeter thyroid nodules.

There were 66 (18.9%) nondiagnostic nodules, 46 (13.2%) benign nodules, 34 (9.7%) AUS/FLUS nodules, 12 (3.4%) FN/SFN nodules, 91 (26.1%) SM nodules, and 100 (28.7%) malignant nodules (Figure 3). The adequate sample rate was 81.1% (283/349).

Surgical resection was performed in 66.8% (233 of 349) of the cases. There were 206 malignant nodules and 27 benign nodules. Out of the 283 cases with diagnostic results, 73.5% (208 of 283) of cases underwent surgery. Among them, 192 were malignant and 16 were benign (Table 1). Among the 25 cases with nondiagnostic results, there were four nodular goiters, seven follicular adenomas, and 14 papillary carcinomas (Table 1).

### 3.2. Adequate Sample Rate in Relation to Nodule-Related Factors

In regard to nodule size, the mean size of the 349 nodules was 7.5 ± 1.9 mm (range 3–10 mm); 31.5% (110 of 349) of nodules measured between 3 and 6 mm and 68.5% (239 of 349) of nodules measured between 7 and 10 mm. In univariate analysis, the adequate sample rate increased with larger nodules; it was 72.7% (80 of 110) for 3–6-mm nodules and 84.9% (203 of 239) for 7–10 mm nodules (*P*=0.007) (Table 2). The adequate sample rate in nodules without calcification was significantly higher than that of nodules with either microcalcification (90.6% vs. 81.3%; *P*=0.035) or macrocalcification (90.6% vs. 63.9%; *P* > 0.001). Furthermore, there was also significant difference between nodules with microcalcification and macrocalcification (*P*=0.005) (Table 2). In regard to other nodule-related factors, there was no significant difference in the adequate sample rate on the basis of nodule location, composition, shape, margin, and echogenicity.

### 3.3. Adequate Sample Rate in Relation to Technical Factors

For the FNAB, a 23-gauge needle was routinely used (75.1% (262 of 349)) and a 22-gauge needle was selectively used (24.9% (87 of 349)). The adequate sample rate was 82.1% (215 of 262) for biopsies performed with a 23-gauge needle and 78.2% (68 of 87) for biopsies performed with a 22-gauge needle. There was no significant difference between the 23- and 22-gauge needle (*P*=0.421). The adequate sample rate of the specimens sampled by using the parallel needle path was significantly higher than that using the perpendicular needle path (85.0% vs. 71.8%; *P*=0.004) (Table 3). No significance was found in the adequate sample rate in relation to an individual radiologist (*P*=0.331).

### 3.4. Multivariate Statistical Analysis

Because some factors may explain other factors, leading to indirect bias, odds ratios (OR) were estimated for diagnostic yield by using multivariate logistic regression. The significant candidate variables were (a) nodule size, (b) calcification, and (c) needle path.

Regarding nodule size, there was an approximately 3.0 times higher likelihood of having a diagnostic result (*P* < 0.001; OR = 2.96; 95% confidence interval (CI): 1.63–5.37) with each increase in nodule size subgroup (3–6 mm, 7–10 mm) (Table 4). For calcification, the highest adequate sample rate correlated with a nodule without calcification, which was approximately 5.3 times more likely than a nodule with macrocalcification to achieve a diagnosis (*P* < 0.001; OR = 5.31; 95% CI: 2.22–12.68) (Table 4). For needle path, the use of a parallel needle path was about 1.8 times more likely than with the use of a perpendicular needle path to achieve a diagnosis (*P*=0.044; OR = 1.84; 95% CI: 1.02–3.33) (Table 4).

## 4. Discussion

Thyroid nodules are common, and thyroid cancer is currently the fifth most common cancer in women [22]. With the improvement of high-frequency ultrasound, the detection rate of subcentimeter thyroid nodules has been significantly improved. The management of thyroid nodules should consider various factors, and cytological diagnosis of FNAB is the most important determinant in decision making [23]. At our institution, all patients underwent US assessment of the thyroid nodules according to the Thyroid Imaging Reporting and Data System (TI-RADS). Patients with subcentimeter thyroid nodules with suspicious US features (TI-RADS category 4A and higher) were recommended for US-guided FNAB. The previous studies have reported that FNAB is still an effective tool for subcentimeter nodules [7, 10, 13, 24]. However, one of the limitations of FNAB is nondiagnostic results due to insufficient sampling. Our results revealed that the adequate sample rate was 81.1%, and 66 nodules (18.9%) yielded insufficient specimens on initial US-guided FNAB, which was similar to that reported in previous studies [7, 13, 16].

Nodule size is a determining factor for the adequate sample rate. The present study revealed that there was an increase in adequate sample rate as thyroid nodules become larger, which may be related to the difficulty in targeting smaller nodules. The adequate sample rate for the 7–10 mm nodules was significantly higher than that for the 3–6 mm nodules. Moon et al. [16] found that with the increase of nodule size, the inadequate sample rate decreased, and the inadequate sample rate was 20.8% for the 2–6 mm nodules and 15.3% for the nodules larger than 6 mm to 10 mm in diameter (*P*=0.007). Kim et al. [13] reported a significant difference in the adequate sample rate between the nodules equal to or less than 5 mm and the nodules larger than 5 mm to 10 mm in diameter (*P*=0.014). Contrary to the findings reported above, the study by Zhong et al. [7] showed that the nodule size did not appear to influence adequate sample rate and there was no significant difference between thyroid nodules ≤5 mm (*n* = 56; adequate sample rate, 82.2%) and nodules 6–10 mm (*n* = 136; adequate sample rate, 87.5%); furthermore, there were no statistical differences in the diagnostic sensitivity, specificity, positive predictive value, and negative predictive value of FNAB for thyroid nodules with different sizes. However, Kim et al. [13] found the sensitivity for nodules equal to or less than 5 mm was significantly lower than that for the nodules larger than 5 mm to 10 mm. Dong et al. [8] reported that the nodules equal to or less than 5 mm with unclear borders were prone to yielding false positive results.

As for calcification, our study found that the inadequate sample rate of the nodules with macrocalcification was significantly higher than that of nodules with microcalcification or no calcification, which is in accordance with the result obtained by Choi et al. [25]. However, Grani et al. [26] reported that neither micro- nor macrocalcification increased the risk of inadequate sampling. Our results suggested that the highest adequate sample rate correlated with the nodules without calcification, which was approximately 5.3 times more likely to achieve a diagnosis as compared to the nodules with macrocalcification. We hypothesize that the high inadequate sample rate of nodules with macrocalcification may be related to the acquisition of limited solid areas of tumor cells during FNAB. First, it may be difficult to penetrate dense calcification with a fine needle. Second, due to strong posterior acoustic shadowing, the internal content and posterior edge of the nodule cannot be viewed, and US has a limitation in the evaluation of a nodule with macrocalcification. Therefore, the ultrasound features of macrocalcification cannot exclude the presence of a solid component hiding behind the posterior acoustic shadowing. We suggest radiologists should carefully analyze the US or CT imaging of nodules with calcification and choose best location from which to get the sample.

Our results indicated that the use of parallel needle path for biopsying small (≤10 mm) thyroid nodules resulted in a higher adequate sample rate. To our knowledge, only one study has compared the effects of parallel and perpendicular techniques on specimen adequacy from thyroid nodules and reported that the use of parallel needle path significantly decreased the inadequate specimens compared to use of perpendicular needle path; however, there were only 80 nodules in this study [27]. Our clinical observation revealed that if the needle is parallel to the probe, the location of the tip and the entire pathway of the needle can be seen from the skin puncture to the nodule. The result that the parallel needle path approach enhances the specimen adequacy of FNAB depends also on better real time monitoring of the needle tip during the aspiration. However, if the needle is perpendicular to the probe, the needle tip is viewed only as a hyperechoic focus when it enters the nodule, and localization of the needle tip becomes more difficult.

Factors such as nodule location, composition, shape, margin, and echogenicity do not appear to affect the adequate sample rate. As for composition, we expected solid thyroid nodule to be a factor associated with high adequate sample rate. Choi et al. [25] reported that cyst-dominant nodules had a significantly higher inadequate sample rate than solid or solid dominant nodules. However, Grani et al. [26] reported that mixed nodules were more frequently diagnostic. In our study, there was no significant difference in the adequate sample rate between solid and mixed nodules. One possible reason for the result may be that the number of mixed lesions was only 20. Of the 20 mixed nodules, 19 were predominantly solid nodules and one was predominantly cystic nodule. We believe that subcentimeter thyroid nodules are less likely to develop cystic degeneration.

There were several potential limitations to our study. First, our study was of retrospective design. Second, there may be a selection bias, nodules were solid, micro- and macrocalcification very frequent, and the sample size of the mixed nodules and benign nodules is relatively small. Finally, the radiologists may choose different paths to puncture for a particular nodule, and furthermore, according to the professional level, the preference of the individual radiologist for a needle path is difficult to assess.

In conclusion, nodule-related and technical factors affecting adequate sample rate with US-guided FNAB of subcentimeter thyroid nodules were identified. The adequate sample rate was related to nodule size, calcification, and needle path. The results of our study revealed that the adequate sample rate was higher in larger nodules, in nodules without calcification, and upon use of the parallel needle path for biopsy.

## Figures and Tables

**Table 1:** Histologic diagnosis after subsequent surgery in 233 nodules.

Diagnosis at FNAB	No. of cases	Surgical diagnosis
Nondiagnostic	25	Four nodular goiters, seven follicular adenomas, 14 papillary carcinomas
Benign	6	One adenomatous hyperplasia, two thyroiditis, three nodular goiters
AUS/FLUS	4	Two follicular adenomas, two adenomatous hyperplasias
FN/SFN	7	One papillary carcinoma, six follicular adenomas
SM	91	91 papillary carcinomas
Malignant	100	Two medullary carcinoma, 98 papillary carcinomas

**Table 2:** Adequate sample rate in relation to nodule-related factors.

Variable	No. of adequate sampling cases	Total no. of cases	Adequate sample rate (%)	*P* value
Nodule size (mm)				0.007
3–6	80	110	72.7	
7–10	203	239	84.9	

Nodule location				0.916
Right	141	172	82.0	
Isthmus	8	10	80	
Left	134	167	80.2	

Composition				0.312
Solid	269	329	81.8	
Mixed	14	20	70	

Shape				0.244
Ovoid to round	84	110	76.4	
Taller than wide	56	65	86.2	
Irregular	143	174	82.2	

Margin				0.589
Defined	100	121	82.6	
Ill-defined	183	228	80.3	

Echogenicity				0.705
Hypoechoic	248	304	81.6	
Isoechoic	25	33	75.8	
Hyperechoic	10	12	83.3	

Calcification				＜0.001
None	96	106	90.6	
Microcalcification	148	182	81.3	
Macrocalcification	39	61	63.9	

^a^A statistically significant difference in diagnostic yield between nodules without calcification and nodules with either microcalcification (*P*=0.035) or macrocalcification (*P* < 0.001) and nodules with microcalcification and macrocalcification (*P*=0.005).

**Table 3:** Adequate sample rate in relation to technical factors.

Variable	No. of adequate sampling cases	Total no. of cases	Adequate sample rate (%)	*P* value
Biopsy needle gauge				0.421
22	68	87	78.2	
23	215	262	82.1	

Needle path				0.004
Perpendicular	74	103	71.8	
Parallel	209	246	85.0	

Radiologist				0.331
A	94	115	81.7	
B	99	128	77.3	
C	90	106	84.9	

**Table 4:** Adequate sample rate according to various related factors evaluated by multivariate logistic regression.

Variable	Adequate sample rate (%)	*P* value	OR	95% CI
Nodule size (mm)				
3–6	72.7 (80/110)	Reference	1.0	
7–10	84.9 (203/239)	<0.001	2.96	1.63–5.37

Calcification				
None	90.6 (96/106)	<0.001	5.31	2.22–12.68
Microcalcification	81.3 (148/182)	0.001	3.20	1.61–6.37
Macrocalcification	63.9 (39/61)	Reference	1.0	

Needle path				
Perpendicular	71.8 (74/103)	Reference	1.0	
Parallel	85.0 (209/246)	0.044	1.84	1.02–3.33

## Data Availability

The data that support the findings of this study are available from the corresponding author upon reasonable request.
